# Hypertensive disorders of pregnancy and risk of Alzheimer’s disease, vascular dementia, and other related dementia

**DOI:** 10.1093/geroni/igab046.2462

**Published:** 2021-12-17

**Authors:** Karen Schliep, Yue Zhang, Joanne Tschanz, Jennifer Majersik, Julio Facelli, Michael Varner

**Affiliations:** 1 University of Utah, Salt Lake City, Utah, United States; 2 Utah State University, Logan, Utah, United States

## Abstract

Several recent studies have examined whether hypertensive disorders of pregnancy (HDP) are associated with an increased risk for Alzheimer’s disease (AD) and other related dementias (RD) with conflicting findings. Limitations to prior studies include lack of assessing risk by dementia subtype, inadequate sample sizes, and not fully exploring the role of mid-life factors. We performed a retrospective matched cohort study among women with >1 singleton pregnancy (1939–2013) using the Utah Population Database. HDP-exposed women (n=19,989) were one-to-two matched with unexposed women (n=39,679) by 5-year age groups, year of childbirth (within 1 year), and parity (1, 2, 3, 4, ≥5) at the time of the pregnancy. HDP pregnancies were complicated by preeclampsia (62%), gestational hypertension (34%), and eclampsia (4%). Women with a history of HDP had a higher hazard of all-cause dementia (HR=1.37; 95% CI: 1.26, 1.50) compared to women without a history of HDP after adjustment for maternal age, year of childbirth, and parity. The hazard doubled after additionally accounting for pre-pregnancy BMI (HR=2.31; 95% CI: 1.24, 4.32). Stratifying by dementia subtype, we found HDP to be associated with a higher hazard of vascular dementia (HR=1.64; 95% CI: 1.19, 2.26) and other related dementia (HR=1.49; 95% CI: 1.34, 1.65) but not Alzheimer’s disease (HR=1.04; 95% CI: 0.87, 1.24) after accounting for competing risks. Mid-life hypertension and stroke were found to have the greatest mid-life impact, mediating 43% and 41% of dementia risk, respectively, highlighting women who may most benefit from close surveillance and early preventive and clinical interventions.

